# Case Study: Impacts of Air-Conditioner Air Supply Strategy on Thermal Environment and Energy Consumption in Offices Using BES–CFD Co-Simulation

**DOI:** 10.3390/s23135958

**Published:** 2023-06-27

**Authors:** Luhan Wang, Guannan Li, Jiajia Gao, Xi Fang, Chongchong Wang, Chenglong Xiong

**Affiliations:** 1 School of Urban Construction, Wuhan University of Science and Technology, Wuhan 430065, China; 2 Anhui Province Key Laboratory of Intelligent Building and Building Energy-Saving, Anhui Jianzhu University, Hefei 230601, China; 3 Key Laboratory of Low-Grade Energy Utilization Technologies and Systems (Chongqing University), Ministry of Education of China, Chongqing University, Chongqing 400044, China; 4 State Key Laboratory of Green Building in Western China, Xi’an University of Architecture & Technology, Xi’an 710055, China; 5 Hubei Provincial Engineering Research Center of Urban Regeneration, Wuhan University of Science and Technology, Wuhan 430065, China; 6 College of Civil Engineering, Hunan University, Changsha 410082, China

**Keywords:** air conditioner, building energy simulation (BES), computational fluid dynamics (CFD), air distributions, thermal environment, energy consumption

## Abstract

Due to energy constraints and people’s increasing requirements for indoor thermal comfort, improving energy efficiency while ensuring thermal comfort has become the focus of research in the design and operation of HVAC systems. This study took office rooms with few people occupying them in Wuhan as the research object. The EnergyPlus-Fluent co-simulation method was used to study the impact of 12 forms of air distribution on the thermal environment and air-conditioner energy consumption. The results indicate that 3 m/s supply air velocity and 45° supply air angle are more suitable for the case model in this study. The EnergyPlus-Fluent co-simulation method used in this paper provides a reference for the study of indoor environments in offices with few people occupying them.

## 1. Introduction

The building sector accounts for approximately 30% of total global energy consumption [1,2,3]. Approximately 60% of building energy consumption is used by heating, ventilation and air-conditioning (HVAC) systems [4,5]. Humans spend about 90% of their time working and living indoors which as a result requires higher indoor thermal comfort [6]. To improve building energy efficiency and ensure indoor thermal comfort, the past decades have witnessed a rapid development in many advanced control-related methods such as building energy consumption prediction [7,8,9,10], fault diagnosis [11,12,13] and model interpretation [14,15,16] of HVAC systems, faulty sensor calibration [17,18,19], etc.

Specifically, many researchers have studied indoor thermal comfort and the energy saving potential of air-conditioning systems. For split air-conditioners, Amoabeng et al. [20] studied the effect of a set-point temperature on the thermal comfort and energy-saving potential for office buildings in hot–humid climates. Kong et al. [21] developed an occupancy-based intelligent building control strategy to improve building energy efficiency as well as occupant comfort. With the help of an EnergyPlus–Fluent coupling method, Shan et al. [22] determined the optimal temperature constants for each partition to solve the problem of an uneven thermal environment between partitions and even provide a low-energy thermal comfort environment for large, open office spaces. Amasyali et al. [23] proposed a data-driven method to assess the potential for improving occupant behavior while reducing energy consumption and increasing comfort. Revathi et al. [24] discovered the optimal sequence of ventilation set-points by considering the meteorological conditions affecting the environment in the greenhouse as a means of ensuring thermal comfort and reducing energy consumption. Ismail et al. [25] established an individualized, cooling radiant compartment and used computational fluid dynamics (CFD) to investigate its effect on the thermal comfort of the cooling load in the office space.

Although the aforementioned energy saving and indoor thermal comfort studies considered many factors including occupant factors, indoor set-points, and re-planed spatial distributions, they still rely heavily on efficient indoor air distributions to supply comfort and high-quality air to air-conditioned spaces [26]. For the energy efficiency and thermal comfort of a target classroom environment, Taylor et al. [27] investigated the effect of three indoor environmental parameters including air velocity, humidity, and air temperature. Wan et al. [28] discussed the influence on ventilation performance of human factors caused by the supply vane angle and supply air temperature. Jagadeesh et al. [29] used CFD as a stimulus of the existing ventilation, no ventilation, and additional ventilation in the classroom and the results when compared with experimental data showed that the room temperature would be at a comfortable level with the provision of additional vents in the classroom. To sum up, it is of great significance to optimize the air-conditioning system design to improve the energy efficiency while ensuring thermal comfort.

Building energy simulation (BES) programs have been widely used as the bases to improve the energy performance of building systems [30]. Commonly, a multi-zone modelling approach is employed by many BES programs such as TRNSYS [31], EnergyPlus [32], and ESP-r [33], etc. However, the modelling approach treats each building zone as a single node with uniform distribution of temperature, pressure, and concentration. As a result, it would be difficult to understand and visualize the heat flow patterns and air distributions introduced by the building space configuration via these BES models [34,35]. On the contrary, CFD programs such as Fluent enables efficient and detailed calculations of air distributions and flow patterns [36]. However, Fluent requires detailed boundary conditions for the CFD solution which could be very difficult to define by itself [37]. Fluent alone can also hardly be applied to estimate energy consumption with high accuracy. Obviously, the two types of programs, BES and CFD, are supplementary to each other in building energy and environmental simulation applications. To overcome the drawbacks of the two programs, researchers have attempted to combine them in a coupled way to simultaneously achieve fast and accurate evaluations of the building energy and indoor environment. With concerns about the gap in computational speed between BES and CFD, Zhai et al. [37] proposed static coupling and dynamic coupling strategies in simulations of offices and indoor race tracks, which demonstrated that the coupling strategy could improve the accuracy of energy consumption prediction. After comparison between BES alone and a quasi-dynamic coupling strategy, Bandara et al. [38,39] found that the co-simulation showed a higher accuracy for building energy prediction. Du et al. [40] optimized the placement of indoor temperature sensors by means of a BES–CFD co-simulation. Yamamoto et al. [41] obtained the temperature distribution in the examined space using the static coupling approach. In Hadavi’s [42] study, BES–CFD co-simulation was used to investigate the effects of urban buildings on the microclimate and cooling system efficiency. Kong et al. [43] applied a coupling strategy for indoor airflow based on real-time information exchange to the simulation of offices with ventilation and radiant floor-cooling systems. Shen et al. [44] used CFD–EnergyPlus dynamic coupling to analyze the effects of community morphology and wind environment on energy consumption for different types of buildings. From the current literature, it can be found that: (1) As for the simulation investigations of air-conditioner energy efficiency and indoor thermal comfort, most published papers have focused on a single simulation method (either BES or CFD, independently). Although some have applied BES–CFD to co-simulate the indoor comfort and air-conditioner energy efficiency in large space buildings, quite a few used the BES–CFD co-simulation method for small office-building rooms by considering the indoor thermal environment and building energy efficiency simultaneously. (2) Not only the locations of the air return and supply temperatures, but also the air supply velocity and angle of air conditioners have impacts on indoor air distributions (temperature and velocity fields), which would further affect the thermal comfort and air-conditioner energy consumption.

Considering the aforementioned issues, this study attempts to quantitatively investigate the air-conditioner energy consumption and building thermal comfort in small-space office room by combining the EnergyPlus with Fluent via external coupling strategy. A case study of office rooms with few people occupying them located in Wuhan was employed for the experimental test and simulation model validation. The impacts of the air-conditioner air supply strategy on the thermal environment and energy consumption in office rooms are fully evaluated by taking air velocity and supply angles into account using a BES–CFD co-simulation. This study considered four different air supply angles of 30°, 45°, 60°, and 90° from the vertical plane and three different air velocities of 3 m/s, 4 m/s, and 5 m/s. After experimental and numerical investigation on the case study, this paper provides certain references for optimizing the air supply strategy of the air conditioner in office rooms with few people occupying them.

## 2. Methodology

### 2.1. Coupling Methods of EnergyPlus and Fluent

The BES–CFD co-simulation method is of two types: internal and external coupling [45]. The internal coupling requires that the two programs (EnergyPlus and Fluent) share a common server. In Djunaedy’s study [46,47], both of the coupling methods were used to simulate the building energy consumption with concerns about the simulation speed and simulation accuracy. The results revealed that the external coupling showed a much faster modelling speed while the two coupling strategies obtained a similar modelling accuracy. Actually, most current BES–CFD co-simulation studies adopt the external coupling strategy which uses BES to provide CFD with the required real-time boundary conditions while the CFD-calculated heat-related physical quantities are passed to correct realtime BES simulations.

**Figure 1:**
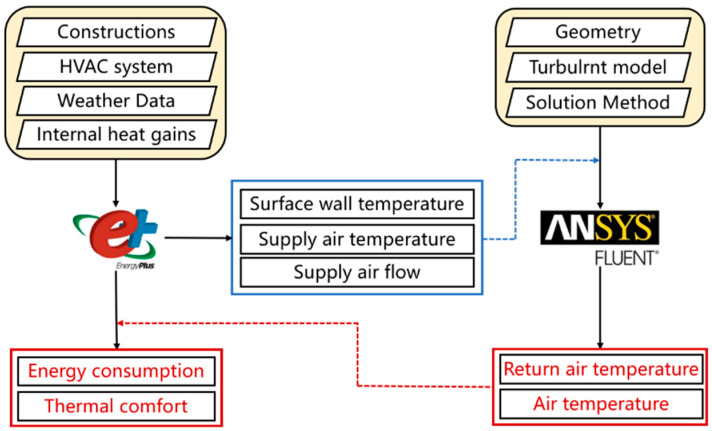
Framework of the external coupling EnergyPlus–Fluent co-simulation in this study.

In this study, EnergyPlus was used to simulate the air-conditioning energy consumption and indoor thermal comfort while Fluent was employed to obtain the indoor air distributions and the entire temperature field of the target office room. Figure 1 shows the simulation framework of the external coupling EnergyPlus–Fluent. On the one hand, EnergyPlus provides necessary boundary conditions to Fluent, including temperature boundaries, air-conditioning outlet flow rate, and air supply temperature. These help Fluent achieve more accurate predictions of the building’s indoor environment. On the other hand, Fluent simulates the indoor air-temperature distributions using the wall temperature boundary from EnergyPlus and the boundary conditions of the air-conditioning air supply outlet. EnergyPlus can obtain the actual temperature of indoor occupied areas through Fluent, resulting in more accurate predictions of air-conditioning energy consumption and indoor thermal comfort.

### 2.2. Thermal Comfort Metrics

This study adopted two thermal comfort evaluation metrics including the predicted mean vote (PMV) and predicted percent dissatisfied (PPD) [48]. PMV represents a predictor of the thermal sensation produced by any given combination of environmental variables. PMV classifies thermal sensation into 7 levels ranging from +3 to −3, namely hot, warm, slightly warm, neutral, slightly cool, cool and cold, as expressed in Equation (1):

(1) $$\begin{array}{ll} PMV & =\left[0.303exp\left(-0.036M\right)+0.0275\right]\\ & \;\times \left\{M-W-3.05\left[5.773-0.007\left(M-W\right)-P_{r}\right]-0.42\left(M-W-58.2\right)\right\}\\ & \;-0.0173M\left(5.867-P_{r}\right)-0.0014M\left(34-t_{air}\right)\\ & \;-3.96\times 10^{-8}f_{cl}\left[{\left(t_{cl}+273\right)}^{4}-{\left(t_{rad}+273\right)}^{4}\right]-f_{cl}h_{c}\left(t_{cl}-t_{air}\right) \end{array}$$

where M is the human energy metabolic rate, W/m^2^; W is the mechanical work performed by the body, W; $P_{r}$ is the partial pressure of water vapor in the air around the body, Pa; $t_{air}$ is the air temperature around the body, °C; $t_{rad}$ is the average radiation temperature of the room, °C; $f_{cl}$ is the ratio of the exterior area of the body when wearing clothes to the surface area of the body when naked; $t_{cl}$ is the temperature of the exterior surface of the clothes, °C; $h_{c}$ is the surface heat transfer coefficient, W/(m^2^·K).

Even in an environment where most people are satisfied, some people may still feel uncomfortable [49]. Hence, practical thermal comfort evaluation usually uses PMV together with PPD. PPD indicates the percentage of dissatisfaction with the environment, as expressed in Equation (2):

(2) $$PPD=100-95exp\left[-\left(0.03353{PMV}^{4}+0.2179{PMV}^{2}\right)\right]$$

In this study, Fluent simulates the indoor air-temperature distribution in an air-conditioned room and transfers the temperatures of the occupants’ working areas to EnergyPlus to estimate the thermal sensation. Thus, the effect of air distributions on the indoor thermal environment can be investigated.

### 2.3. Numerical Simulation Methods

In this study, the Fluent simulation model is developed based on the control equations including mass conservation, momentum conservation, energy conservation equation, turbulent kinetic energy k, and ε equations as follows:

(1) Mass conservation

(3) $$\frac{\partial u_{i}}{\partial x_{i}}=0$$

where $u_{i}$ is the partial velocity in the direction of $x_{i}$, m/s.

(2) Momentum conservation

(4) $$\frac{\partial }{\partial x_{i}}\left(\rho u_{i}u_{j}\right)=-\frac{\partial p}{\partial x_{i}}+\frac{\partial }{\partial x_{j}}\left[\mu \left(\frac{\partial u_{i}}{\partial x_{j}}+\frac{\partial u_{j}}{\partial x_{i}}\right)-\frac{3}{2}\mu \frac{\partial u_{i}}{\partial x_{i}}\delta_{ij}\right]+\rho g_{i}+F_{i}$$

where p is the air static pressure, Pa; ρ is the air density, kg/m^3^; $g_{i}$ is the gravitational acceleration in direction i; $\rho g_{i}$ is the volume force in direction i, N/m^3^; Fi is the source term due to the heat source; μ is the dynamic viscosity, Pa·s.

(3) Energy conservation equation

(5) $$\frac{\partial \left(\rho u_{i}h\right)}{\partial x_{i}}=\frac{\partial }{\partial x_{i}}\left[\left(\frac{\mu }{p_{r}}+\frac{\mu_{t}}{\sigma_{t}}\right)\frac{\partial h}{\partial x_{i}}\right]+S_{h}$$

where $\mu_{t}$ is the turbulent viscosity coefficient, Pa·s; $\mu_{t}=\rho C_{\mu }\frac{k^{2}}{\varepsilon }$; $S_{h}$ is source term of volumetric heat source; $p_{r}$ is the turbulent Prandtl number; h is specific enthalpy of air at constant pressure, J/kg.

(4) Turbulent kinetic energy k equation

(6) $$\frac{\partial \left(\rho k\right)}{\partial t}+\frac{\partial \left(\rho \varepsilon u_{i}\right)}{\partial x_{i}}=\frac{\partial }{\partial x_{j}}\left[\left(\mu +\frac{\mu_{i}}{\sigma_{k}}\right)\frac{\partial k}{\partial x_{j}}\right]+\mu_{t}\frac{\partial u_{i}}{\partial x_{j}}\left(\frac{\partial u_{i}}{\partial x_{j}}+\frac{\partial u_{j}}{\partial x_{i}}\right)-\rho \varepsilon$$

(5) Turbulent kinetic energy ε equation

(7) $$\frac{\partial \left(\rho \varepsilon \right)}{\partial t}+\frac{\partial \left(\rho \varepsilon u_{i}\right)}{\partial x_{i}}=\frac{\partial }{\partial x_{j}}\left[\left(\mu +\frac{\mu_{i}}{\sigma_{s}}\right)\frac{\partial \varepsilon }{\partial x_{j}}\right]+\frac{C_{1s}}{k}\frac{\partial u_{i}}{\partial x_{j}}\left(\frac{\partial u_{i}}{\partial x_{j}}+\frac{\partial u_{j}}{\partial x_{i}}\right)-C_{2s}\rho \frac{\varepsilon^{2}}{k}$$

where model constants are C_1s_ = 1.44, C_2s_ = 1.92, C_μ_ = 0.09, σ_k_ = 1.0, and σ_s_ = 1.3.

For the target-simulated air-conditioned rooms in this study, to simplify the numerical simulation process and the established mathematical model, several assumptions are required as follows: (1) the air is viscous and incompressible, and the air flow is steady-state turbulent; (2) the air conforms to the Boussinesq assumption, and the density variation in the default fluid affects only the buoyancy force; (3) the radiant heat transfer between walls and internal heat sources should be neglected; (4) the target room is assumed to be of good air tightness so the infiltration should be neglected; (5) the effect of air relative humidity on the air distribution in the target room can be neglected.

The Fluent model adopts the RNG k-ε turbulence model which is suitable for characterizing the air-flow field in air-conditioned rooms [50]. Indoor airflow is usually turbulent and is considered incompressible in CFD simulations [51]. The separation solver and the SIMPLEC algorithm are used to solve the control equations.

## 3. Case Study

### 3.1. EnergyPlus Model

**Figure 2:**
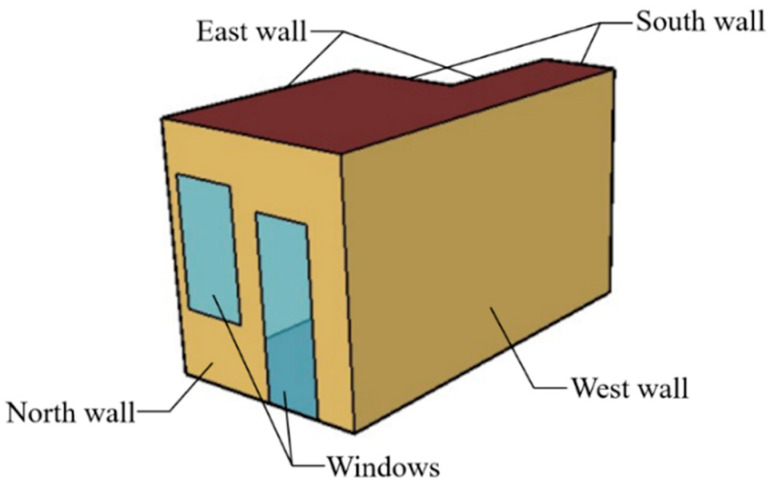
Illustration of the EnergyPlus building model.

In this study, the simulated building is an office room located in Wuhan. The basic room sizes are 4.81 m length, 2.5 m width, and 2.88 m height. The office is divided into two areas: air-conditioned and non-air-conditioned areas. The air-conditioned area is 9.25 m^2^. For the studied air conditioner, the nominal cooling and heating capacity are 3500 W and 4500 W, respectively. Its nominal cooling and heating power are 1110 W and 1500 W, respectively. To obtain the wall surface temperature, supply air volume, and supply air temperature, this study first developed an EnergyPlus model for the separate office, as shown in Figure 2. The north wall is the external wall and the corridor is outside the south wall. The rest are the inner walls adjacent to the rooms with the air conditioning off. The heat transfer coefficients of the exterior wall, exterior window, and the roof are 0.55 W/(m^2^·K), 2.1 W/(m^2^·K), and 0.37 W/(m^2^·K), respectively. The EnergyPlus model uses parameters of the actual experimental air conditioner in the office and the typical meteorological year data of Wuhan are used for modelling. The air conditioner is a constant air volume type working from 09:00 to 17:00 each day. Only one person is simulated in the room with indoor temperature set as 26 °C at heating operations.

### 3.2. Fluent Model

**Figure 3:**
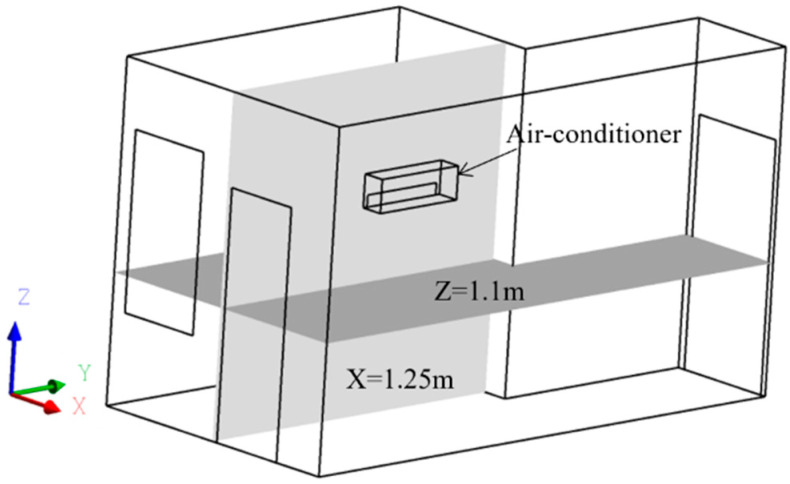
Simplified Fluent model for air conditioner in an office room.

Due to the computer hardware limitations, this study simplified the computational model to simulate the real room. Assume that the person in the room is in a sedentary state. The air conditioner is simplified to a rectangle of 0.82 m × 0.28 m × 0.19 m. The specific layout of the overall model office of the room is shown in Figure 3.

### 3.3. Grid Independence Test

**Figure 4:**
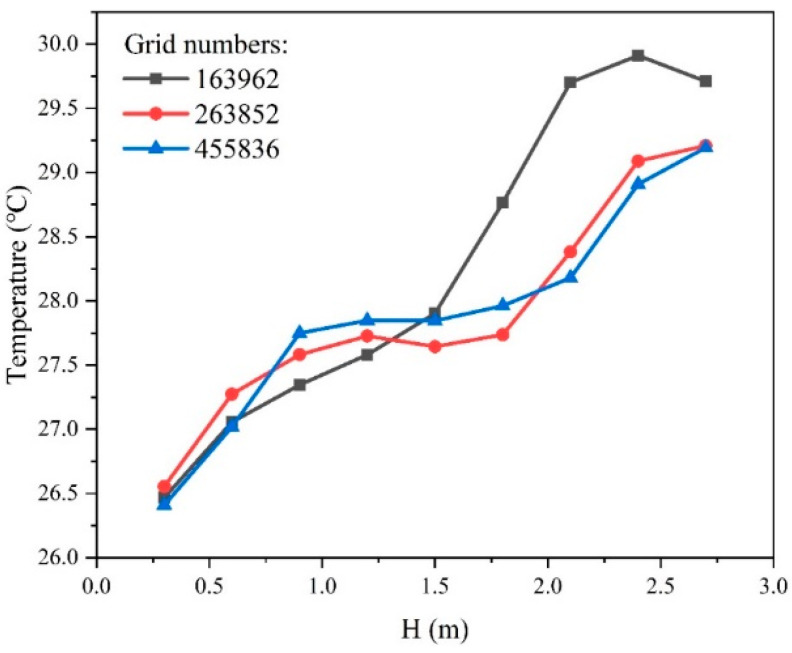
Grid independence test results.

**Figure 5:**
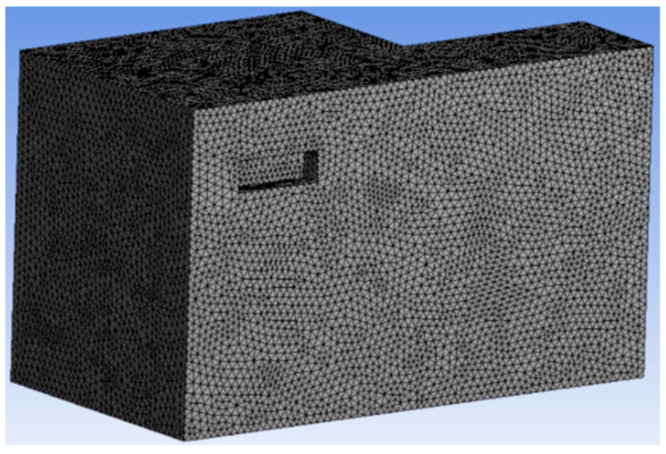
Illustration of grid division of the air-conditioned area.

The grid independence test is conducted to reduce the impact of grids on simulation results. Figure 4 shows the grid independence test results after comparing the temperatures of nine points on the line segment (x, y, z) = (1.25, 2, 0.3–2.7) m under three grid sizes: 160 thousand, 260 thousand, and 450 thousand grid divisions. For the three grid sizes, the temperature variation trends are quite consistent. In particular, the temperature difference between the 260 thousand grid and 450 thousand grid is less than 1%. Therefore, this study chose the grid partitioning method of 260 thousand grid for the Fluent simulation, as shown in Figure 5. The grid size of the supply and return air outlets is 0.05 m while the grid size of other places is 0.07 m.

### 3.4. Boundary Conditions

**Figure 6:**
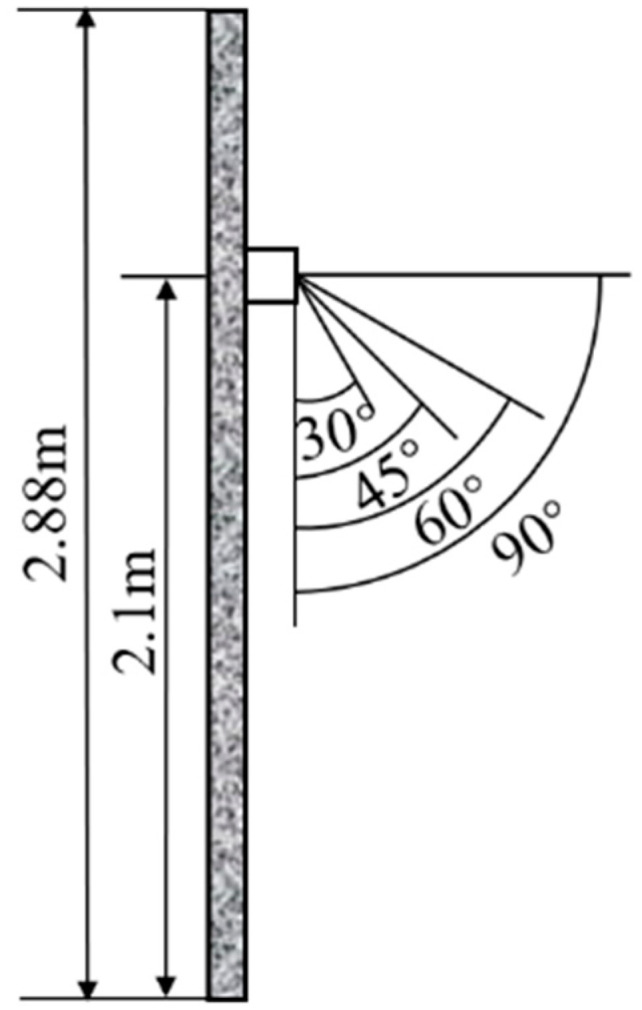
The four different airflow angles considered.

Boundary conditions are another important part of Fluent simulation. Different boundary condition settings can lead to completely different simulation results. In this study, the boundary conditions of Fluent are provided by EnergyPlus. The heating temperature is set as 26 °C in EnergyPlus. Based on the air outlet area in the Fluent model, the three different air supply velocities 3 m/s, 4 m/s, and 5 m/s are air flow volumes of 0.1098 m^3^/s, 0.1464 m^3^/s, and 0.183 m^3^/s, respectively. The air supply temperature and indoor heat source boundary conditions are predicted for the three velocities. The boundary conditions of the co-simulation and the Fluent simulation are summarized in Table 1. The boundary conditions of the Fluent simulation are calculated based on the indoor–outdoor temperature difference and the heat transfer coefficient of the building envelope. Since rooms next to the target experimental room are non-air-conditioned, the heat flow boundary of the inner wall still uses the indoor and outdoor temperature difference. The velocity inlet boundary is used for the air supply outlet. The exit boundary conditions are free outflow. In addition to air supply velocities and temperatures, four different air supply angles of 30°, 45°, 60°, and 90° are set out in Figure 6.

### 3.5. Experiment Validation

**Figure 7:**
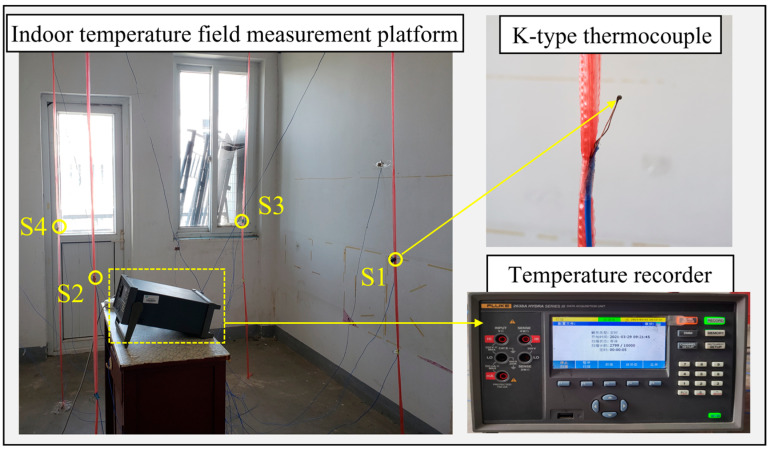
Experimental platform.

**Figure 8:**
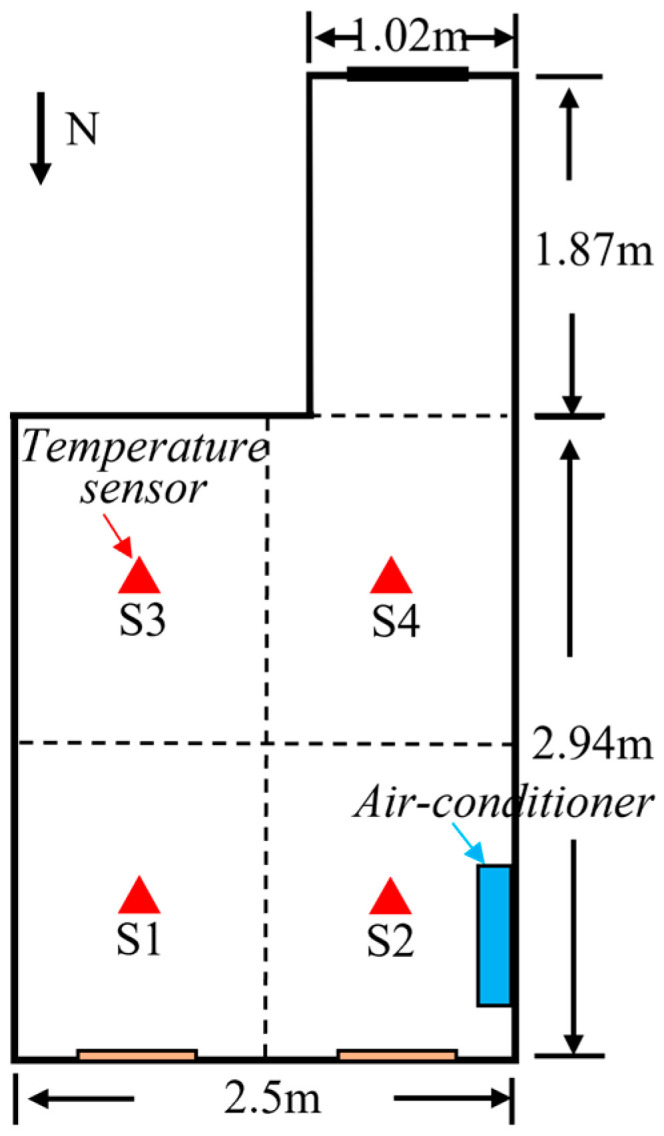
Sensor locations in four sub-zones of the air-conditioned area.

To validate the model simulation accuracy, indoor temperatures were measured and collected from the office room. The K-type thermocouples were used. The temperature recorder measured the range from −270 °C to 1372 °C with a temperature measurement accuracy of 0.6 °C. Figure 7 shows the four sensors were arranged at a head height of approximately 1.1 m in the office when the person was sitting still. The measurements were performed in winter. The temperature acquisition interval was 1 min. The air conditioner operated from 09:00 to 17:00 and the temperature was fixed at 26 °C. The experimental air-conditioner air-velocity block was used to measure the experimental air velocity (about 3 m/s in this study). The air supply angle was first set as about 50° horizontally facing downward. The personnel occupancy area is divided equally into four zones. For each zone, a sensor measurement point is arranged in the center. Locations of the temperature sensors are depicted in Figure 8.

**Figure 9:**
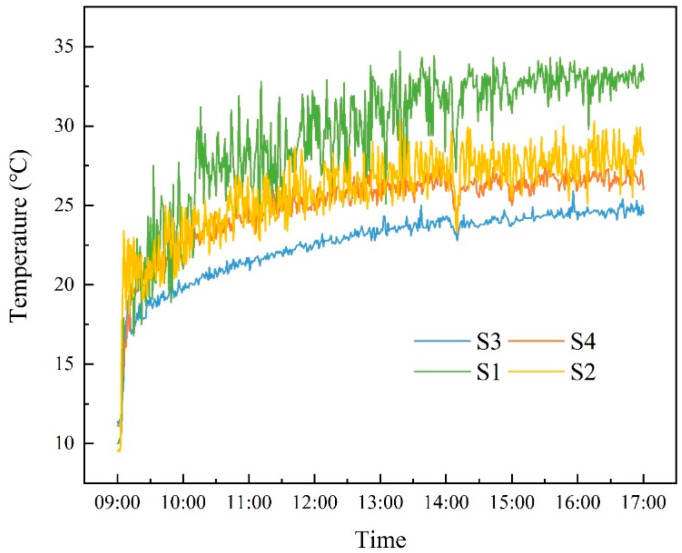
The temperature profiles measured by four sensors.

Figure 9 shows the indoor temperature variations in the four temperature sensors. S1 and S2 show larger temperature fluctuations than the other two points. This is because the two points are placed on the side of the air-conditioner outlet. Air outflow from the air conditioner would lead to great temperature fluctuations in airflow near the measurement point. After the air conditioner began to work at 09:00, the air temperature rose rapidly. At around 13:00, the indoor temperatures of the four sub-zones tended to be steady which shows a much lower temperature increase than the previous 4 h.

**Figure 10:**
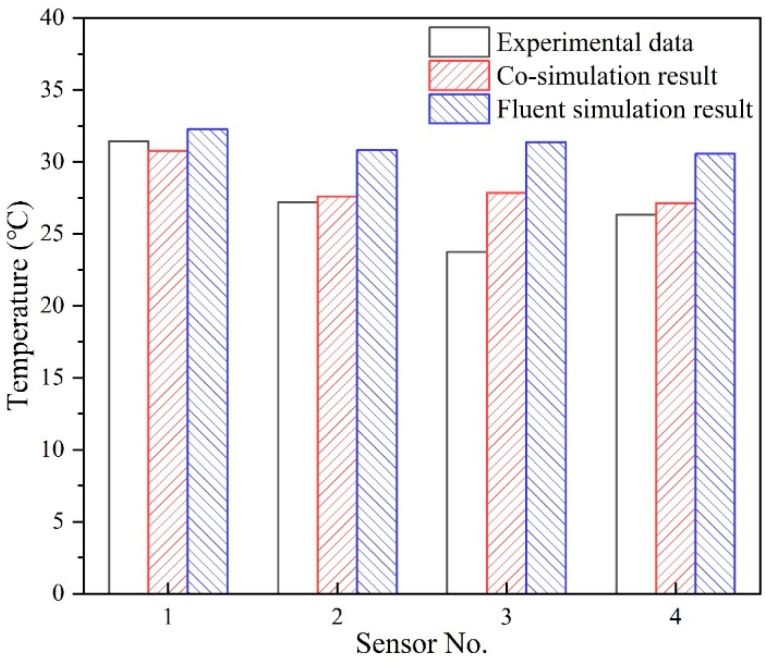
Comparisons between experimental data and simulation results.

In order to verify the co-simulation results, the data exchange was chosen at 13:00 when the data started to stabilize. An example of 3 m/s supply air-speed case was used for model validation. The temperatures of the four positions were derived from the co-simulation and the Fluent simulation. The simulated data were compared with the experimental data as shown in Figure 10. Considering the temperature fluctuations in experiments, the average temperature values from 13:00 to 13:59 were compared with the simulation results. The results indicate that co-simulation results are closer to the experimental data than the simulation results for Fluent alone.

## 4. Results and Discussion

### 4.1. Indoor Temperature Analysis

**Figure 11:**
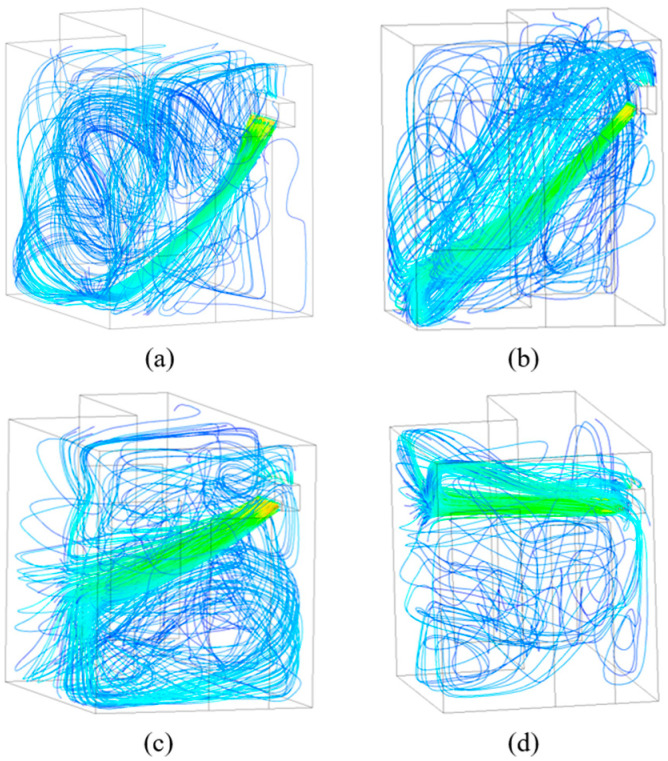
Streamline of four wind directions: (**a**) 3 m/s-30°; (**b**) 3 m/s-45°; (**c**) 3 m/s-60°; (**d**) 3 m/s-90°.

Taking the 3 m/s supply air velocity as an example, the streamlines of the four wind directions are shown in Figure 11a–d. To better compare the effects of different airflow organization on indoor thermal comfort and air-conditioning energy consumption, cross-sections with X = 1.25 m and Z = 1.1 m were selected for further comparisons. The positions of the cross-sections in the model are shown in Figure 3.

#### 4.1.1. Z = 1.1 m Cross-Sectional Temperature Cloud

**Figure 12:**
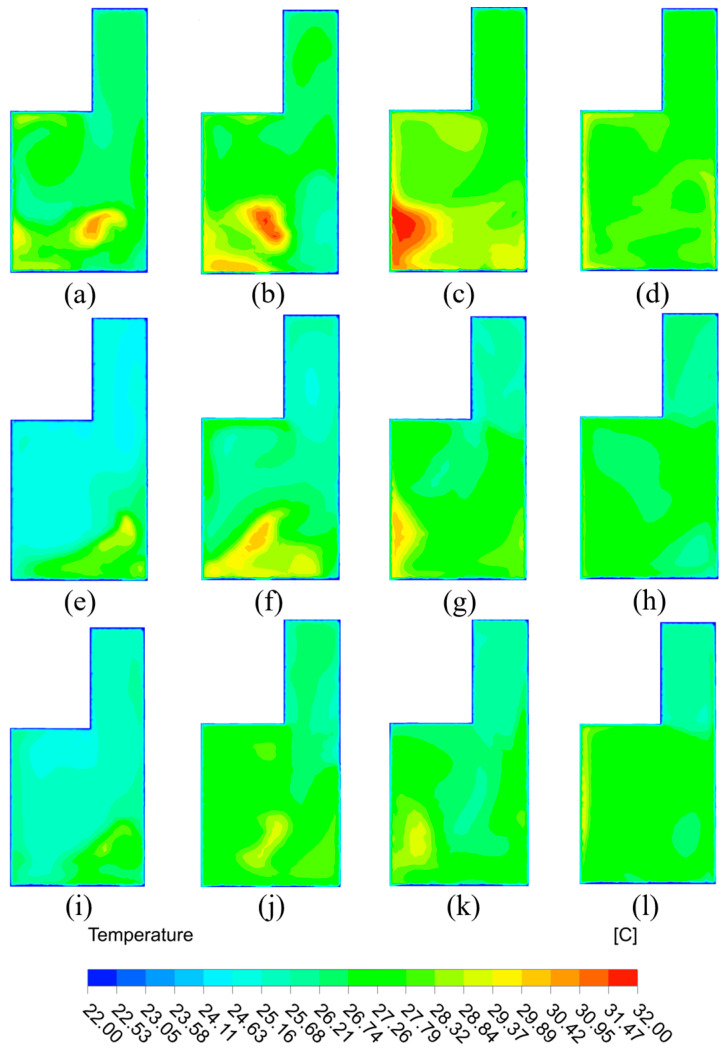
Temperature cloud of Z = 1.1 m section: (**a**) 3 m/s-30°; (**b**) 3 m/s-45°; (**c**) 3 m/s-60°; (**d**) 3 m/s-90°; (**e**) 4 m/s-30°; (**f**) 4 m/s-45°; (**g**) 4 m/s-60°; (**h**) 4 m/s-90°; (**i**) 5 m/s-30°; (**j**) 5 m/s-45°; (**k**) 5 m/s-60°; (**l**) 5 m/s-90°.

Figure 12a–l show the temperature distribution of the Z = 1.1 m cross section with four different air supply angles when air velocities are 3 m/s, 4 m/s, and 5 m/s. When the supply air velocity is 3 m/s, obvious temperature gradients can be seen in the cross section with air supply angles of 30°, 45°, and 60°. This is because the air supply path for the three air-supply angles can pass through the cross section of Z = 1.1 m. However, when the air supply angle increases to 90°, the air supply path may fail to pass through the Z = 1.1 m section. As a result, the temperature distribution of the section is more uniform for the 90° angle. If the supply air velocity grows to 4 m/s, the temperature distribution of the cross-section is more uniform for air supply angles of 30° and 90°. The temperature gradient on the cross-section at 45° and 60° air supply angle is smaller than that at the 3 m/s supply air velocity. The possible reason is that when air is supplied with angles of 45° and 60°, the supply air is much closer to the room center. This contributes to the heat exchange between supply warm air and indoor air. Hence, the overall temperature of the cross section tends to be higher for air supply angles of 45° and 60°. At 5 m/s supply air velocity, the temperature of the cross-section was relatively uniform at all four air supply angles. The air supply angles of 45° and 60° still had temperature gradients. The temperature at the location where the air supply path intersects the cross section is higher than the other locations of the cross section; however, the temperature difference is not significant.

To sum up, the temperature distribution is more uniform at 30° and 90° air supply angles for the cross-section of Z = 1.1 m. For both 45° and 60° air supply angles at the same cross-section, the temperature gradient is relatively more obvious. The gradient decreases as the supply air velocity increases. In terms of supply air velocity, larger air velocity could cause increasingly uniform temperature distribution in the cross-section. If the air velocity is concentrated at 3 m/s, an obvious high temperature area appears in the room, as shown in Figure 12a–c.

#### 4.1.2. X = 1.25 m Cross-Sectional Temperature Cloud

**Figure 13:**
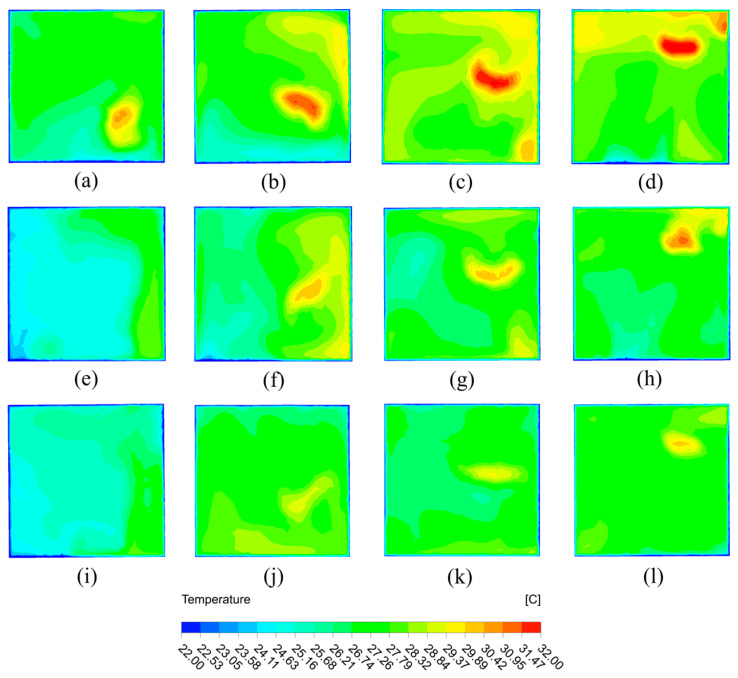
Temperature cloud of X = 1.25 m section: (**a**) 3 m/s-30°; (**b**) 3 m/s-45°; (**c**) 3 m/s-60°; (**d**) 3 m/s-90°; (**e**) 4 m/s-30°; (**f**) 4 m/s-45°; (**g**) 4 m/s-60°; (**h**) 4 m/s-90°; (**i**) 5 m/s-30°; (**j**) 5 m/s-45°; (**k**) 5 m/s-60°; (**l**) 5 m/s-90°.

Figure 13a–l show the temperature distribution of the X = 1.25 m cross section with four different air supply angles when air velocities are 3 m/s, 4 m/s, and 5 m/s. When the 3 m/s supply air velocity is used, there are distinct high temperature areas in the air supply path at different angles. Since the high temperature area is concentrated, significant temperature differences exist on the cross-section. When the air supply angle is 30°, the temperature gradients are still relatively small on the upper and lower sides of the room. When the air supply angle is 90°, the hot air gathers above the room due to the lower air density, and the temperature gradient on the upper and lower sides of the room tends to be very significant. When the supply air velocity is 4 m/s, the temperature distribution pattern on the cross section is nearly the same as that of the 3 m/s supply air velocity. However, the temperature gradient tends to be smaller for the case of the 4 m/s supply air velocity. The temperature concentration in the high temperature region is not as significant as the case of 3 m/s. The 90° air supply angle does not cause a significant temperature difference in the personnel-working area; however, there is a significant temperature difference in the occupied area under the other three air supply angles. When the supply air velocity is 5 m/s, the temperature distribution within the whole cross-section is very uniform because of the higher air supply velocity. No obvious concentrated area of high temperature appears in the cross-section and the temperature gradient is not apparent.

After a comparison of all 12 groups of airflow organization, it can be found that the most uniform temperature distribution appears in the cast that when the air supply angle is 30°. Since the air supply paths passing through the occupied area at angles of 30°, 45°, and 60°, there are temperature differences in the occupied area under other air supply angles except for the 90° air supply angle. In terms of supply air velocity, the section temperature is most uniform at 5 m/s. At 3 m/s, the hot air is more concentrated above the space, and the temperature differences are obvious between the upper and lower sides of the room.

#### 4.1.3. Measurement Point Temperatures

**Figure 14:**
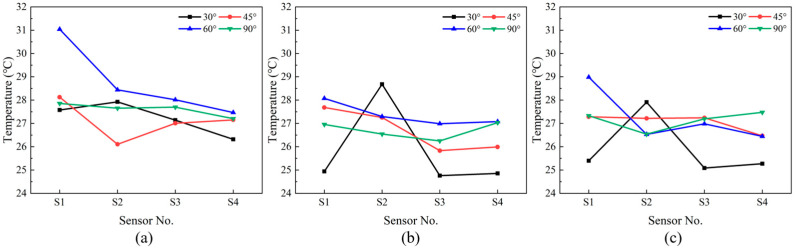
Temperatures of the 4 sensors with different air velocities: (**a**) 3 m/s; (**b**) 4 m/s; (**c**) 5 m/s.

The temperatures of the four sensor locations located around the personnel simulated by the co-simulation under twelve airflow organizations are output as shown in Figure 14a–c. For the three different air supply velocities, temperatures of the four locations are more stable for the air supply angle of 90° with little difference and a lower overall temperature. This is because that the air supply path is further away from the four sensor measurement points at this angle. The temperature of the measuring point of the S1 sensor is higher than the other three measuring points when the air supply angle is 45° and 60°. This is because the hot air is closer to the S1 position for the two air supply angles. The highest temperature is at the location of S2 when the air supply angle is 30° since it is influenced by the air supply path. The concentration of high temperature areas around S2 caused the temperature at S2 to be higher than the other three locations. The four sensor temperatures are averaged to represent the average temperature in the occupied area at a cross-section of Z = 1.1 m. This temperature was inputted into EnergyPlus to calculate the thermal comfort in the occupied area and the air-conditioning energy consumption with different air distributions.

### 4.2. Thermal Comfort Analysis

**Figure 15:**
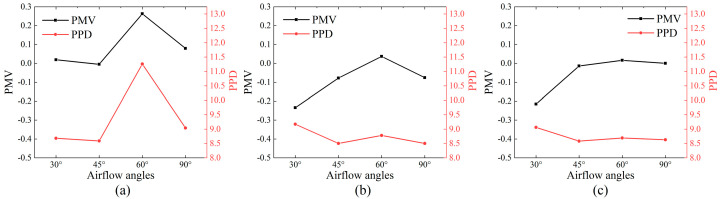
Average PMV−PPD for different supply air velocities: (**a**) 3 m/s; (**b**) 4 m/s; (**c**) 5 m/s.

The average PMV-PPD values for personnel-occupied areas with different air distributions are shown in Figure 15a–c. Overall, PMV ranges from −0.3 to 0.3 with the maximum difference being less than 1. This indicates no significant differences of thermal comfort. For PPD, it reaches the maximum at a supply angle of 60° with a wind speed of 3 m/s. However, for larger wind speeds of 4 m/s and 5 m/s, PPD achieves the maximum at a supply angle of 30°. However, the PPD difference under other airflow organization conditions is not significant. From the perspective of thermal comfort, the case model is most suitable for a 45° air supply angle.

### 4.3. Energy Consumption Analysis

**Figure 16:**
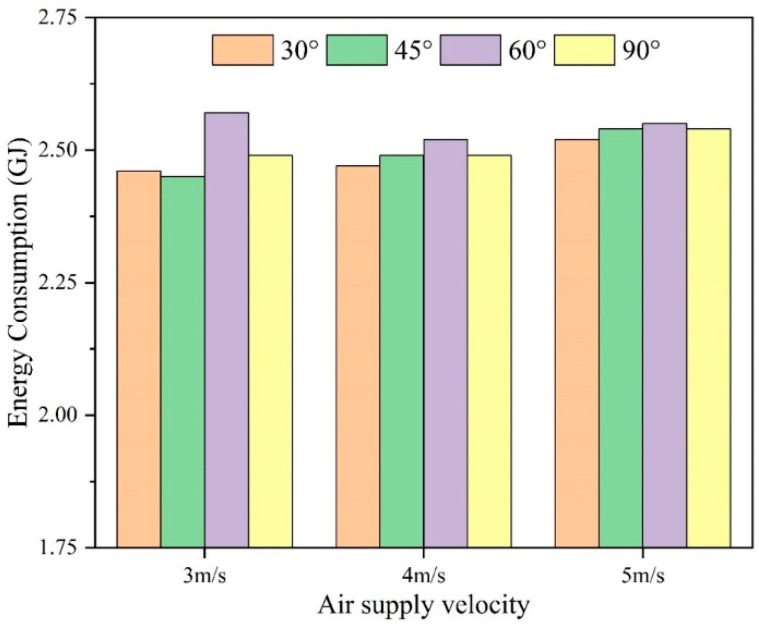
Air-conditioning energy consumption under different airflow organizations.

For heating seasons from November to March in the year, the air-conditioning energy consumption under different air flow patterns is shown in Figure 16. The air-conditioning energy consumption is highest at a 60° supply angle in all four wind directions. At 5 m/s wind speed, the difference in energy consumption between the four wind directions is smaller than the difference in energy consumption between the four wind directions with air velocities of 3 m/s and 4 m/s. Overall, the air conditioner consumes the least energy at a wind speed of 3 m/s. At a wind speed of 5 m/s, the air conditioner consumes the most energy, which is related to the energy consumption of the fan.

## 5. Conclusions

In this paper, a BES–CFD co-simulation method (EnergyPlus–Fluent) was developed and applied to investigate the effects of various air supply velocities and angles on the indoor thermal environment and energy efficiency of air conditioners in the office room. To validate the developed co-simulation method, an independent office room in Wuhan was employed as an example case study. The EnergyPlus–Fluent model was validated by experimental data with relatively higher modelling accuracy than Fluent alone. After model validation, this study simulated a total of three groups of air velocities (3 m/s, 4 m/s, and 5 m/s) and four groups of air supply angles (30°, 45°, 60°, and 90°) at air conditioning outlets for further analysis. The effects of various air distributions on indoor temperature distribution and air-conditioning energy consumption were investigated. The conclusions are as follows:

(1) The EnergyPlus–Fluent model can be used to conduct a building energy and environmental simulation simultaneously for small-space office building rooms with few people occupying them. The BES–CFD co-simulation was validated to outperform the simulation using Fluent alone in terms of modelling accuracy.
(2) For the small-space office room, a slightly higher air supply velocity would contribute to better temperature distribution. However, if both thermal comfort and energy consumption are considered simultaneously for the target room in the case study, 3 m/s supply air velocity and 45° air supply angle should be recommended.

This study successfully applies the EnergyPlus–Fluent simulation method to simultaneously conduct a building energy and environmental simulation for small-space office building rooms with few people occupying them. The results provide a reference for analyzing the air supply mode of air conditioning in office rooms from the perspective of the temperature field using the BES–CFD co-simulation method, rather than simply from the perspective of only using the BES simulation method. Although the co-simulation model was validated using experimental data with higher accuracy for a target office room, future validation works of BES–CFD co-simulation are still required for more complicated indoor environments (such as temperature, humidity, etc.) and more types of buildings with different enclosure structures, shapes, sizes, and air-conditioning systems.

## Figures and Tables

**Table 1:** Boundary conditions under different air supply conditions.

Name	Co-Simulation			CFD
Air supply velocity	3 m/s	4 m/s	5 m/s	3 m/s
Air supply temperature	35.33 °C	32.89 °C	31.51 °C	35.33 °C
Ceiling	18.87 °C	18.92 °C	18.92 °C	4.74 W/m^2^
Floor	18.09 °C	18.11 °C	18.11 °C	Adiabatic
East wall	19.93 °C	20.05 °C	20.05 °C	15.73 W/m^2^
West wall	19.93 °C	20.05 °C	20.05 °C	15.73 W/m^2^
South wall	19.93 °C	20.06 °C	20.06 °C	15.73 W/m^2^
North wall	18.09 °C	18.14 °C	18.14 °C	6.95 W/m^2^

## Data Availability

Not applicable.

## Nomenclature

HVAC	Heating, ventilation, and air conditioning
CFD	Computational fluid dynamics
BES	Building energy simulation
M	Human energy metabolic rate
PMV	Predicted mean vote
PPD	Predicted percent dissatisfied
W	Mechanical work performed by the body
$P_{r}$	Partial pressure of water vapor in the air around the body
$t_{air}$	The air temperature around the body
$t_{rad}$	The average radiation temperature of the room
$f_{cl}$	The ratio of the exterior area of the body when wearing clothes to the surface area of the body when naked
$t_{cl}$	The temperature of the exterior surface of the clothes
h	Specific enthalpy of air at constant pressure
$h_{c}$	Surface heat transfer coefficient
$u_{i}$	The partial velocity in the direction of $x_{i}$
p	Air static pressure
ρ	Air density
$g_{i}$	The gravitational acceleration in direction i
$\rho g_{i}$	The volume force in direction i
$F_{i}$	The source term due to the heat source
μ	Dynamic viscosity
$\mu_{t}$	Turbulent viscosity coefficient
$S_{h}$	The source term of volumetric heat source
$p_{r}$	Turbulent Prandtl number
$T_{x}$	The simulated temperature of the measurement point
$T_{e}$	Experimental measurement temperature
